# Display of a thermostable lipase on the surface of a solvent-resistant bacterium, *Pseudomonas putida *GM730, and its applications in whole-cell biocatalysis

**DOI:** 10.1186/1472-6750-6-23

**Published:** 2006-04-19

**Authors:** Heung-Chae Jung, Seok-Joon Kwon, Jae-Gu Pan

**Affiliations:** 1 National Research Laboratory of Microbial Display, GenoFocus, Inc., 461-58 Jeonmindong, Yusong, Daejeon 305-811, Republic of Korea; 2 Systems Microbiology Research Center, Korea Research Institute of Bioscience and Biotechnology (KRIBB), 52 Oundong, Yusong, Daejeon 305-333, Republic of Korea; 3 Present address: Department of Chemical and Biological Engineering, Rensselaer Polytechnic Institute, Troy, NY 12180-3590, USA

## Abstract

**Background:**

Whole-cell biocatalysis in organic solvents has been widely applied to industrial bioprocesses. In two-phase water-solvent processes, substrate conversion yields and volumetric productivities can be limited by the toxicity of solvents to host cells and by the low mass transfer rates of the substrates from the solvent phase to the whole-cell biocatalysts in water.

**Results:**

To solve the problem of solvent toxicity, we immobilized a thermostable lipase (TliA) from *Pseudomonas fluorescens *on the cell surface of a solvent-resistant bacterium, *Pseudomonas putida *GM730. Surface immobilization of enzymes eliminates the mass-transfer limitation imposed by the cell wall and membranes. TliA was successfully immobilized on the surface of *P. putida *cells using the ice-nucleation protein (INP) anchoring motif from *Pseudomonas syrinage*. The surface location was confirmed by flow cytometry, protease accessibility and whole-cell enzyme activity using a membrane-impermeable substrate. Three hundred and fifty units of whole-cell hydrolytic activity per gram dry cell mass were obtained when the enzyme was immobilized with a shorter INP anchoring motif (INPNC). The surface-immobilized TliA retained full enzyme activity in a two-phase water-isooctane reaction system after incubation at 37°C for 12 h, while the activity of the free form enzyme decreased to 65% of its initial value. Whole cells presenting immobilized TliA were shown to catalyze three representative lipase reactions: hydrolysis of olive oil, synthesis of triacylglycerol and chiral resolution.

**Conclusion:**

*In vivo *surface immobilization of enzymes on solvent-resistant bacteria was demonstrated, and appears to be useful for a variety of whole-cell bioconversions in the presence of organic solvents.

## Background

Biocatalysis that exploits the catalytic activities of enzymes has emerged as a promising approach to chemical synthesis of novel and industrially significant compounds [1,2]. Enzymes can catalyze reactions exhibiting enantioselectivity and regioselectivity under appropriate conditions. Despite the tremendous repertoire of enzyme reactions in nature, their applications are limited because of enzyme availability (screening, overexpression and purification), substrate range and operational stability [1]. Enzyme immobilization can often be used to improve product separation, operational stability and reusability. However, during immobilization, enzyme activity and natural properties are lost and the reusability of the immobilized enzyme is also limited. As these issues can be overcome by using whole cells, whole-cell biocatalysis has been widely adopted for the commercial synthesis of a variety of compounds, from bulk chemicals to valuable pharmaceuticals [2,3] or for bioremediation [4]. Compared with isolated enzymes, whole-cell biocatalysts can be much more readily and inexpensively prepared on an industrial scale. Owing to their diversity and ease of handling, microbial cells have been most commonly utilized for whole-cell biocatalysis.

One of the technical problems in whole-cell biocatalysis is the mass-transfer limitation, since the cell membrane and wall act as a permeability barrier, thus requiring a permeabilization step. Permeabilization in turn may induce other problems, such as enzyme inactivation and the release of cellular components. A more innovative solution to the mass-transfer limitation problem would be to immobilize the target enzyme on the bacterial surface [5,6]. The use of whole cells presenting surface-bound enzymes for biocatalysis (*in vivo *immobilization) may provide an alternative to the traditional way of performing enzymatic reactions [7,8].

*In vivo *immobilization of foreign proteins on the surfaces of Gram-negative bacteria is usually accomplished through fusion of the target protein with endogenous outer membrane proteins such as LamB, PhoE, OmpA, Lpp-OmpA, OprF, OmpC, OmpS, invasin and INP [5,6]. The target proteins are either fused to the N-terminal or C-terminal or inserted into a permissive region. We have developed a stable procedure using the ice-nucleation protein (INP) [7], an outer membrane protein from *Pseudomonas syringae *that catalyzes the formation of ice crystals in super-cooled water [9]. Foreign proteins were surface-immobilized not only by whole INP but also by truncated versions [10,11]. INP immobilization technology is now well established for whole-cell biocatalysis [7], vaccine development [10] and combinatorial screening of enzyme libraries [12,13].

In whole-cell biocatalysis, process productivity has been frequently limited because substrates or products of interest are sparingly soluble in water and/or toxic to the producer microorganism [14]. Two-phase water-solvent systems provide an alternative methodology for performing efficient bioconversion because they increase the solubilities of hydrophobic substrates or products and/or change the kinetic equilibrium, enhancing productivity [15,16]. The solvents might be chosen according to their polarities as quantified by a logarithmic parameter, log P_o/w_, where P_o/w _is the partition coefficient of a given solvent in an equimolar mixture of octanol and water [17]. Hydrophobic solvents (log P_o/w _> 5) are usually considered to comply with biocompatibility criteria.

Bacteria resistant to organic solvents allow a new degree of freedom in coping with toxic solvents [18]. They can survive and even grow normally in pure organic solvents that are considerably less lipophilic than octane, even toluene (log P_o/w _= 2.7) or heptanol (log P_o/w _= 2.4) [19,20]. Since the initial discovery of a toluene-tolerant *Pseudomonas putida *[21], other strains of *P. putida *[22] and other species of the genus *Pseudomonas *have been reported [23]. Furthermore, solvent tolerance has recently been found in strains of the Gram-positive bacteria *Bacillus *[24,25] and *Rhodococcus *[26].

In this study, we immobilized an enzyme on the surfaces of solvent-resistant bacteria to provide whole-cell biocatalysts for biotransformation reactions in the presence of organic solvents. To do this, a thermostable lipase (TliA) from *Pseudomonas fluorescens *SIK W1 [27] was expressed on the surface of *Pseudomonas putida *GM730 [28] using INP fusion, and the *in vivo *immobilized enzyme was shown to serve as a whole cell biocatalyst for three representative lipase reactions: lipid hydrolysis in a two-phase reaction, triacylglycerol synthesis without water and chiral resolution in organic solvent. The lipase (TliA) from *P. fluorescens *SIK W1, the optimum pH and temperature of which are 8.5 and 45~55°C, respectively [29], was selected owing to its usefulness in a variety of reactions [30]. Its stability to organic solvents was not investigated, though it can be inferred from the available literature that lipases are generally stable in organic solvents, with a few exceptions of stimulation or inhibition [31].

## Results and discussion

### In vivo immobilization of lipase on the Pseudomonas putida cell surface

In our previous work, the thermostable lipase TliA was expressed on the *Escherichia coli *surface and used for whole-cell biocatalysis in olive oil hydrolysis and for screening improved enzyme variants after mutation [13]. To express TliA on the surface of a solvent resistant bacterium, *Pseudomonas putida *GM730 [28], we used the same strategy to construct the INPNC-TliA and INP-TliA fusion proteins, the expression of which was directed from the surface display vectors pJHC13 and pJHC14. Their constructions were based on a broad host-range vector, pRK415 [32].

**Figure 1:**
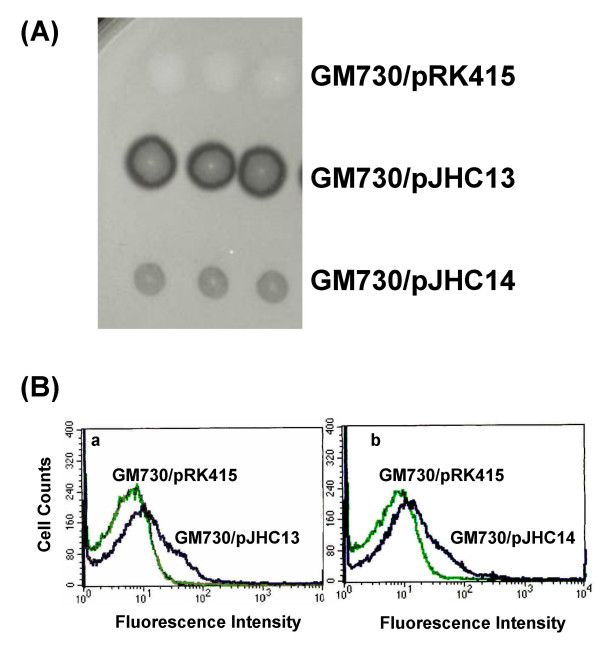
(A) Halo-formation around the colonies of recombinant *P. putida *GM730 cells. (B) Probing the surface display of the lipase by flow cytometry using rabbit-anti-TliA antibodies and fluorescein-labeled ant-rabbit IgG antibodies. a. Histogram of GM730/pRK415 (control cells) and GM730/JHC13 (INPNC-TliA displaying cells). b. Histogram of GM730/pRK415 (control cells) and GM730/JHC143 (INP-TliA displaying cells).

Active lipase expression in *P. putida *GM730 was verified by halo formation around the colonies on a tributyrin (1% v/v) emulsion agar plate (Figure 1A). Colonies of control cells (*P. putida *GM730/pRK415) did not form halos during incubation for 24 h at 25°C, indicating that the host cells did not secrete any lipolytic enzymes into the growth medium. Halos formed around the GM730/pJHC13 and GM730/pJHC14 colonies. The cells expressing INPNC-TliA (GM730/pJHC13) formed much larger halos than those expressing INP-TliA (GM730/pJHC14).

To confirm immobilization of the lipase on the *P. putida *GM730 cell surfaces, whole-cell enzyme activities were measured with *p*-nitrophenyl palmitate (pNPP), which does not enter the cells. Lipase activities of 350 and 163 units per g dry cell mass were obtained when PBS-washed GM730/pJHC13 and GM730/pJHC14 cells, respectively, were induced with 1 mM IPTG. The corresponding lipase activities in the culture supernatant did not exceed 30 units/g dry cell mass, indicating that there was no release of lipase into the medium during cell growth and induction of the fusion proteins. The shorter anchoring motif, INPNC, mediated the immobilization of more lipases on *P. putida *GM730 cells than did INP. Total INPNC was 3~4 times more highly expressed than INP, as determined by levansucrase and CMCase fusion experiments in our laboratory (data not shown). Thus, the amount of lipase targeting to the outer membrane was higher (normally two-fold higher) with INPNC than with INP.

The presence of TliA on the *P. putida *cell surfaces was further verified by flow cytometry as described previously [13]. As shown in Figure 1B, IPTG-induced and PBS-washed cells were probed with primary rabbit polyclonal antibodies reactive to TliA, and thereafter fluorescently stained with a fluorescein (FITC)-labeled secondary antibody. The data show that the negative cells (GM730/pRK415) did not react with the anti-TliA antibodies, but the positive cells (GM730/pJHC13 and GM730/pJHC14) reacted, confirming that the lipase was immobilized on the *P. putida *GM730 surface.

### Stability of in vivo immobilized lipase in a two-phase, water-organic solvent system

**Figure 2:**
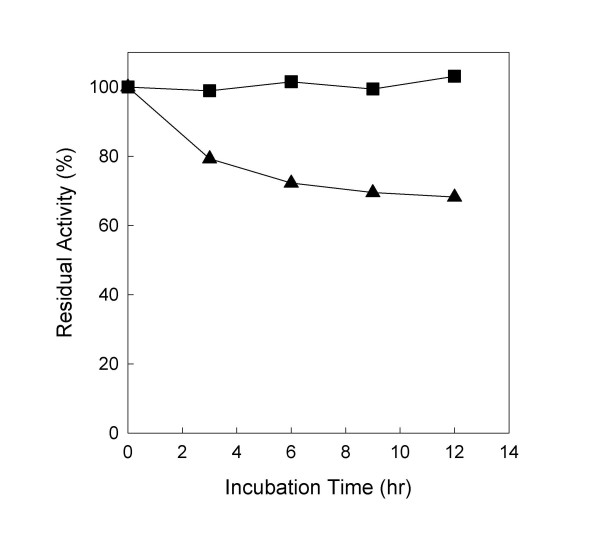
Stability of lipase displayed on the surface of *P. putida *GM730/pJHC13 cells (■) and free form lipase (▲) during incubation at 37°C in a two-phase water-isooctane reaction system. Residual lipase activities were measured spectrophotometrically with pNPP as a substrate and calculated by assuming the initial activity was 100%.

We compared the *in vivo *immobilized lipase with free lipase in a two-phase water-organic solvent system. As described previously [33], isooctane was selected as the solvent phase to dissolve hydrophobic substrates in the two-phase bioconversion system. As shown in Figure 2, whole-cell lipase activity was maintained for 12 h in the water-isooctane system, whereas the activity of the free enzyme was decreased to 65% of its initial value. One possible explanation for the stabilization of lipase in the water-isooctane reaction system might be the effect of immobilization on the bacterial cell surface. Lee *et al*. demonstrated that surface-immobilized lipases were stable to heat and organic solvents when the lyophilized cells were incubated at high temperature or in organic solvents [34,35]. Similar results were obtained from lipases immobilized on yeast surfaces [36]. These results suggest that surface immobilization of lipases, like immobilization of enzymes on synthetic resins, is a method of choice for stabilizing them against heat or organic solvents.

### Hydrolysis of olive oil in a two-phase fermentation system

**Figure 3:**
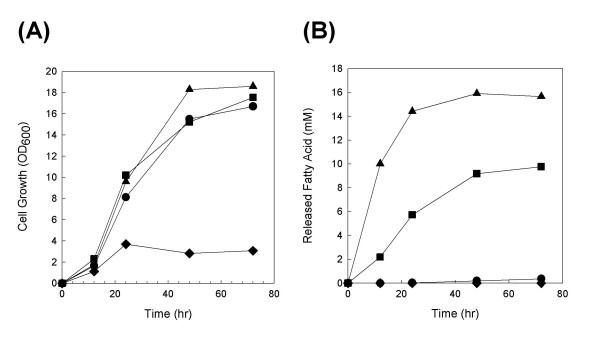
Lipid hydrolysis by surface-displayed lipase in two-phase water-isooctane fermentation system. (A) Cell growth of, (B) fatty acids released by, *P. putida *GM730 cells containing pRK415 (●), pJHC13 (▲), pJHC14 (■) and *E. coli *JM109/pJHC11 (◆).

We tested the TliA-decorated *P. putida *cells as a whole-cell biocatalyst for hydrolyzing olive oil in a two-phase water-solvent fermentation system. For this reaction, 10% (w/v) olive oil dissolved in isooctane was vigorously mixed with an aqueous solution containing complex growth medium. Overnight cultures of recombinant *P. putida *cells and *E. coli *JM109/pJHC11 cells were inoculated into the mixtures. Cell growth in the aqueous phase and hydrolysis of olive oil in the isooctane phase were monitored. As shown in Figure 3, all the GM730 cells grew normally in the two-phase system. In the isooctane phase, the concentrations of fatty acids produced by the hydrolysis of olive oil by GM730/pJHC13 and GM730/pJHC14 cells were 15.9 mM and 9.8 mM, respectively, while no hydrolysis was detected in the control cells (GM730/pRK415). *E. coli *JM109/pJHC11 cells with immobilized INPNC-TliA were unable to grow normally or hydrolyze olive oil. Clearly, *E. coli *cells could not grow in the biphasic water-isooctane fermentation system owing to the toxicity of isooctane, but *P. putida *grew normally in that system. These results suggest that GM730 cells are highly stable to isooctane and grow in a biphasic reaction system, and that the surface-immobilized lipase was active and available for hydrolyzing olive oil in this system.

### Synthesis of triacylglycerol in isooctane

**Figure 4:**
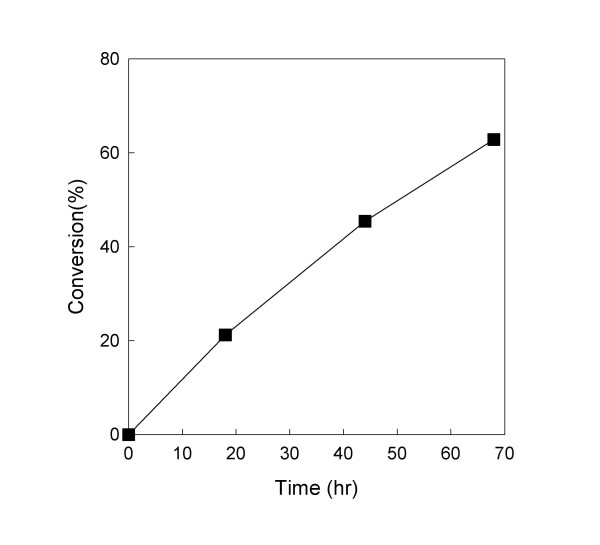
Synthesis of triacylglycerol by lipase displayed on the *P. putida *GM730/pJHC13 cell surfaces in the isooctane solvent reaction system at 37°C. Conversion yield during incubation is shown.

We also investigated whether *P. putida *cells carrying immobilized lipases can used to perform a synthetic reaction, the reverse of lipase-catalyzed hydrolysis, in an organic solvent. Triacylglycerol synthesis in isooctane was used as a model. We used GM730/pJHC13 as a whole-cell catalyst because its lipase activity was higher than that of GM730/pJHC14. *P. putida *GM730/pJHC13 cells were prepared by cultivation at 30°C, induction with 1 mM IPTG and harvesting by centrifugation. The cells were washed with PBS and finally air-dried. Air-dried cells (100 mg) were dispersed in 10 ml reaction mixture. As shown in Figure 4, a 65% (w/w) yield was obtained from 100 mM of glycerol and 10 mM oleic acid in isooctane after 68 h reaction. These results suggest that *in vivo *immobilized lipase on the surface of solvent-resistant bacteria can be used for efficient triglyceride synthesis in an organic solvent.

### Chiral resolution of racemic 4-nitrophenyl 2-phenylpropionate in a biphasic reaction system

**Figure 5:**
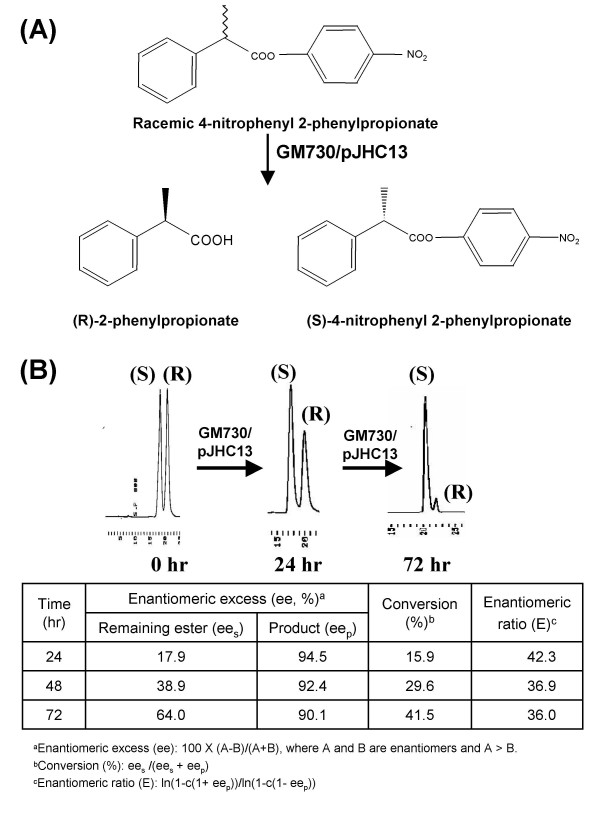
Chiral resolution of racemic 4-nitrophenyl 2-phenylpropionate (NPPP) in two-phase water-isooctane reaction system. (A) Schematic diagram for chiral resolution of NPPP. (B) Chromatograms and time dependence of reaction by *P. putida *GM730/pJHC13 cells over 72 h.

Recently, many enantiomerically pure compounds have been produced by regio- and enantio-selective reactions with lipase, such as the kinetic resolution of 1-phenylethanol or α-methylene β-lactams, and dynamic kinetic resolution of hemiaminals and cyanohydrin esters [37,38]. In many cases, however, lipases have been used in immobilized form, which is expensive and laborious and represents an obstacle to broadening the use of enzymatic processes. To improve the cost-efficiency of chiral resolution reactions, the use of lipases immobilized on the surfaces of *E. coli *[35,39,40] and *Saccharomyces cereviae *[36] has been proposed. In this study, we illustrated a further use of *in vivo *immobilized lipases: to achieve enantioselective chiral resolution of a racemic mixture of *p*-nitrophenyl 2-phenylpropionate (NPPP) (Fig. 5A), especially in a two-phase aqueous-organic solvent system, since optically pure 2-phenylpropionic acid has been widely used as a chiral building block in fine chemical synthesis. Isooctane was used as the solvent phase for dissolving substrate and product. In the reaction system, 50 mM racemic NPPP was dissolved in 10 ml isooctane, and GM730/pJHC13 cells, washed and prepared as described above, were suspended in 10 ml PBS.

After 72 h in the two-phase water-isooctane system, the enantiomeric excesses of the surviving *p*-nitrophenyl 2-phenylpropionate and the product (R) 2-phenylpropionic acid were 64.0% and 90.1%, respectively (Fig. 5B). The percentage conversion was 41.5% with an enantiomeric ratio of 36. The time course of the reaction is also shown in Fig. 5B. Although the percentage conversion and the enantiomeric ratio were not very high for resolution of p-nitrophenyl 2-phenylpropionate, this result is sufficient to indicate that surface-immobilized lipases on solvent-resistant bacteria have potential for whole-cell resolution of racemic mixtures of chiral compounds in organic solvents. We did not try to optimize the reaction itself further, but as described previously [13], we are now evaluating the surface immobilization system in solvent-resistant bacteria for screening a TliA library in order to obtain lipase mutants with higher chiral selectivity and stability in organic solvents. According to a recent report [41], higher productivity could be achieved in fine chemical synthesis by the partitioning of substrates and products in two-phase systems. Thus, if in the future we use more hydrophilic solvents with higher NPPP solubility, we can improve the conversion yield.

## Conclusion

We demonstrate the immobilization of a thermostable lipase from *Pseudomonas fluorescens *in an active form on the cell surface of *Pseudomonas putida*, a solvent-resistant bacterium, and show that the enzyme catalyzes reactions in the presence of an organic solvent. This *in vivo *immobilized lipase was stably maintained in a water-isooctane system without being released from the cell surface. Two-phase fermentation for lipid hydrolysis was achieved by growing the cells in a mixture of water and solvent phases containing culture medium and olive oil, respectively. Moreover, the *in vivo *immobilized lipase could be used for lipid synthesis and chiral resolution. Because the *in vivo *immobilized lipase can be prepared easily by simple cultivation and separation of the cells, no additional steps for the purification and stabilization (immobilization) are required and can feasibly be modified by rational *de novo *design of the enzyme. Thanks to the advantages of solvent-resistant bacteria as expression hosts for surface immobilization of enzymes, they can be widely applied in whole-cell biocatalytic processes for producing bulk bio/chemicals to fine chemicals. Moreover, a library of enzymes on the surfaces of solvent-resistant bacteria can also provide a high-throughput screening tool for directed evolution of enzymes that will become more stable and active in toxic organic solvents. As described elsewhere [13], we are evaluating this technique to evolve the lipase for chiral selectivity and transesterification in organic solvents.

## Methods

### Bacteria and growth conditions

*E. coli *JM109 (*rec*A1 *supE*44 *endA*1 *hsdR*17 [r_k_^- ^m_k_^+^] *gyrA*96 *rel*A *thi *"Δ[*lac-pro*AB]/F' [*traD*36 *proAB*^+ ^*lacZ*ΔM15]) was used as a host for plasmid construction. An organic solvent-resistant bacterium, *Pseudomonas putida *GM730 [28], was employed for the surface immobilization of lipase and used for whole-cell biocatalysis in organic solvents. *E. coli *and *P. putida *were grown in Luria-Bertani (LB) medium (0.5% yeast extract, 1% tryptone, 0.5% NaCl) at the desired temperatures. Ampicillin (100 μg/ml) or tetracycline (15 μg/ml) was added to the medium to select recombinant cells.

### Construction of plasmids and recombinant *P. putida *cells

The surface immobilization vectors for the lipase, TliA, from *Pseudomonas fluorescens *SIK W1 were derived from pJHC11 and pJHC12, which encode the INP-TliA and INPNC-TliA fusion proteins [13]. For expression in *P. putida *GM730, a broad host-range vector, pRK415, was used [32]. A 2.6 kb DNA fragment containing the *tac *promoter and the gene encoding INPNC-TliA obtained from pJHC11 by *Bam*HI-*Hind*III treatment was inserted into pRK415 treated with the same enzyme, creating pJHC13, which directed expression of the INPNC-TliA fusion protein with 1 mM isopropylthio-β-D-galactopyranoside (IPTG). To generate pJHC14, directing expression of the INP-TliA fusion protein, the same subcloning strategy was applied using pJHC12. An 8 kb DNA fragment containing the *tac *promoter and the gene encoding INP-TliA obtained from pJHC12 by *Bam*HI-*Hin*dIII treatment was inserted into pRK415. The plasmids were introduced into *P. putida *GM730 cells by conjugal transfer with a helper plasmid, pRK2013 [42]. Briefly, overnight cultures of donor (JM109/pJHC13 or JM109/pJHC14), acceptor (GM730) and helper (HB101/pRK2013) cells were inoculated into the main culture. When the cultures reached optical density 0.8 (600 nm), 1 ml of the cells were harvested, washed twice with 0.9% (w/v) saline and resuspended in 200 μl saline. The washed acceptor, helper and donor cells were sequentially dropped and dried on LB agar plate. Finally, the plates were incubated at the growth temperature (30°C) of the acceptor cells. After 8~10 h, the cells were collected and resuspended in sterile saline, then spread on a selective agar plate containing ampicillin and tetracycline. The newly formed colonies of *P. putida *GM730 were verified for presence of pJHC13 and pJHC14.

### Flow cytometry

An overnight culture of recombinant *P. putida *cells was transferred to fresh LB medium and grown to OD_600nm _≈ 0.4. Synthesis of recombinant proteins was induced with 1 mM IPTG and incubation was continued for an additional 6 h at 30°C. The surface localization of the recombinant lipase was confirmed by flow cytometric analysis. For immunofluorescence staining, 10^10 ^cells were harvested and washed three times with PBS. The washed cells were resuspended in 1 ml PBS containing 1% skim milk and rabbit anti-TliA antibody (1:1,000) and incubated on ice for 1 h. After washing three times with PBS, the cells were incubated with FITC-conjugated anti-rabbit IgG antibody (1:100) on ice for 1 h. The FITC-labeled cells were examined under a FACScan flow cytometer (Becton Dickinson, Oxnard, CA).

### Hydrolysis of olive oil in a two-phase fermentation system

A two-phase fermentation system, consisting of 10 ml of isooctane to dissolve the olive oil and 10 ml of LB medium for cell growth, was used for whole cell hydrolysis of olive oil. Olive oil (10% w/v) was dissolved in the isooctane phase. Overnight cultures (0.5 ml) of each recombinant *P. putida *cell were inoculated into the two-phase reaction mixtures in 500 ml baffled flasks, which were incubated in a shaking incubator at 30°C. To induce the INP-TliA and INPNC-TliA fusion proteins, 1 mM IPTG was added at the beginning of the culture. To monitor cell growth and olive oil hydrolysis, samples were taken and centrifuged to separate the phases. Optical density (600 nm) for cell growth was measured in aqueous solution samples. Released fatty acids dissolved in the isooctane phase were determined by the cupric acetate method using oleic acid as a reference.

### Synthesis of triacylglycerol in isooctane

*P. putida *GM730/pJHC13 cells induced with 1 mM IPTG in LB medium (50 ml) were harvested at optical density 9.5 (600 nm) by centrifugation and washed with PBS solution. To remove water, the washed cells were air-dried. To synthesize triacylglycerol in an organic solvent, 10 ml of isooctane was used to dissolve glycerol (100 mM) and oleic acid (10 mM), and Na_2_HPO_4 _(0.2 g anhydrous) was added to adjust the pH. To initiate the reaction, 100 mg of air-dried cells were added to the reaction mixture, followed by incubation at 30°C with stirring (200 rpm). The reaction products and substrates were analyzed by HPLC.

### Chiral resolution of racemic 4-nitrophenyl 2-phenylpropionate

Chiral resolution of racemic NPPP was carried out in a two-phase water-isooctane reaction system. Racemic NPPP (50 mM) was dissolved in 10 ml isooctane, and washed GM730/pJHC13 cells, prepared as described above for the synthetic reaction, were suspended in 10 ml PBS. The two solutions were vigorously mixed in a 250 ml baffled flask at 25°C. The reaction products in the isooctane were analyzed by HPLC.

## Authors' contributions

HCJ: preparation of the manuscript, construction of recombinant DNA and strains, probing of display and flow cytometry, hydrolysis of lipid. SJK: triolein synthesis reaction and chiral resolution. JGP: design and conception of project and follow-up discussions of results. All authors read and approved the final manuscript.

## References

[B1] Schoemaker HE, Mink D, Wubbolts MG (2003). Dispelling the myths-biocatalysis in industrial synthesis. Science.

[B2] Schmid A, Dordick JS, Hauer B, Kiener A, Wubbolts M, Witholt B (2001). Industrial biocatalysis today and tomorrow. Nature.

[B3] Ishige T, Honda K, Shimizu S (2005). Whole organism biocatalysis. Curr Opin Chem Biol.

[B4] Timmis KN, Pieper DH (1999). Bacteria designed for bioremediation. Trends Biotechnol.

[B5] Chen W, Georgiou G (2002). Cell-surface display of heterologous proteins: From high-throughput screening to environmental applications. Biotechnol Bioengin.

[B6] Wernerus H, Stahl S (2004). Biotechnological applications for surface-engineered bacteria. Biotechnol Appl Biochem.

[B7] Jung HC, Lebeault JM, Pan JG (1998). Surface display of *Zymomonas mobilis *levansucrase by using the ice-nucleation protein of *Pseudomonas syringae*. Nat Biotechnol.

[B8] Shimazu M, Mulchandani A, Chen W (2001). Simultaneous degradation of organophosphorus pesticides and *p *-nitrophenol by a genetically engineered *Moraxella *sp. with surface-expressed organophosphorus hydrolase. Biotechnol Bioeng.

[B9] Wolber P, Warren G (1989). Bacterial ice-nucleation proteins. Trends Biochem Sci.

[B10] Lee JS, Shin KS, Pan JG, Kim CJ (2000). Surface-displayed viral antigens on Salmonella carrier vaccine. Nat Biotechnol.

[B11] Jung HC, Park JH, Park SH, Lebeault JM, Pan JG (1998). Expression of carboxymethylcellulase on the surface of *Escherichia coli *using *Pseudomonas syringae *ice nucleation protein. Enzyme Microb Technol.

[B12] Kim YS, Jung HC, Pan JG (2000). Bacterial cell surface display of an enzyme library for selective screening of improved cellulase variants. Appl Environ Microbiol.

[B13] Jung HC, Ko S, Ju SJ, Kim EJ, Kim MK, Pan JG (2003). Bacterial cell surface display of lipase and its randomly mutated library facilitates high-throughput screening of mutants showing higher specific activities. J Mol Cat B: Enzymatic.

[B14] Leon R, Fernandes P, Pinheiro HM, Cabral JMS (1998). Whole-cell biocatalysis in organic media. Enzyme Microb Technol.

[B15] Khmelnitsky YL, Rich JO (1999). Biocatalysis in nonaqueous solvents. Curr Opin Chem Biol.

[B16] Angelova B, Schmauder H-P (1999). Lipophilic compounds in biotechnology: interactions with cells and technological problems. J Biotechnol.

[B17] Isken S, de Bont JAM (1998). Bacteria tolerant to organic solvents. Extremophiles.

[B18] de Bont JAM (1998). Solvent-tolerant bacteria in biocatalysis. Trends Biotechnol.

[B19] Kieboom J, Dennis JJ, de Bont JA, Zylstra GJ (1998). Identification and molecular characterization of an efflux pump involved in *Pseudomonas putida *S12 solvent tolerance. J Biol Chem.

[B20] Sardessai Y, Bhosle S (2002). Tolerance of bacteria to organic solvents. Res Microbiol.

[B21] Inoue A, Horikoshi K (1989). A *Pseudomonas *thrives in high concentrations of toluene. Nature.

[B22] Ramos JL, Duque E, Huertas MJ, Haidour A (1995). Isolation and expansion of the catabolic potential of a *Pseudomonas putida *strain able to grow in the presence of high concentrations of aromatic hydrocarbons. J Bacteriol.

[B23] Ogino H, Yasui K, Watanabe F, Ishikawa H (1996). An organic solvent-tolerant bacterium and its organic solvent-stable protease. Ann N Y Acad Sci.

[B24] Matsumoto M, De Bont JAM, Isken S (2002). Isolation and characterization of the solvent-tolerant *Bacillus cereus *strain R1. J Biosci Bioengin.

[B25] Moriya K, Horikoshi K (1993). Isolation of a benzene-tolerant bacterium and its hydrocarbon degradation. J Ferment Bioengin.

[B26] Kyung-Su NA, Kuroda A, Takiguchi N, Kato J, Ikeda T, Ohtake H (2005). Isolation and characterization of benzene-tolerant *Rhodococcus opacus *strains. J Biosci Bioengin.

[B27] Chung GH, Lee YP, Jeohn GH, Yoo OJ, Rhee JS (1991). Cloning and nucleotide sequence of thermostable lipase gene from *Pseudomonas fluorescens *SIK W1. Agric Biol Chem.

[B28] Kim K, Lee S, Lee K, Lim D (1998). Isolation and characterization of toluene-sensitive mutants from the toluene-resistant bacterium *Pseudomonas putida *GM73. J Bacteriol.

[B29] Lee YP, Chung GH, Rhee JS (1993). Purification and characterization of *Pseudomonas fluorescens *SIK W1 lipase expressed in *Escherichia coli*. Biochim Biophys Acta – Lipids Lipid Met.

[B30] Xie ZF (1991). *Pseudomonas fluorescens *lipase in asymmetric synthesis. Tetrahed Asym.

[B31] Gupta R, Gupta N, Rathi P (2004). Bacterial lipases: an overview of production, purification and biochemical properties. Appl Microbiol Biotechnol.

[B32] Keen NT, Tamaki S, Kobayashi D, Trollinger D (1988). Improved broad-host-range plasmids for DNA cloning in Gram-negative bacteria. Gene.

[B33] Lee SY, Rhee JS (1994). Hydrolysis of triglyceride by the whole cell of *Pseudomonas putida *3SK in two-phase batch and continuous reactor systems. Biotechnol Bioengin.

[B34] Lee SH, Choi J-i, Han M-J, Choi JH, Lee SY (2005). Display of lipase on the cell surface of *Escherichia coli *using OprF as an anchor and its application to enantioselective resolution in organic solvent. Biotechnol Bioengin.

[B35] Lee SH, Choi JI, Park SJ, Lee SY, Park BC (2004). Display of bacterial lipase on the *Escherichia coli *cell surface by using FadL as an anchoring motif and use of the enzyme in enantioselective biocatalysis. Appl Environ Microbiol.

[B36] Matsumoto T, Ito M, Fukuda H, Kondo A (2004). Enantioselective transesterification using lipase-displaying yeast whole-cell biocatalyst. Appl Microbiol Biotechnol.

[B37] Turner NJ (2004). Enzyme catalysed deracemisation and dynamic kinetic resolution reactions. Curr Opin Chem Biol.

[B38] Ghanem A, Aboul-Enein HY (2004). Lipase-mediated chiral resolution of racemates in organic solvents. Tetrahedron: Asymmetry.

[B39] Kim JH, Lee CS, Kim BG (2005). Spore-displayed streptavidin: a live diagnostic tool in biotechnology. Biochem Biophys Res Commun.

[B40] Lee SH, Choi JH, Park SH, Choi J-I, Lee SY (2004). Enantioselective resolution of racemic compounds by cell surface displayed lipase. Enzyme Microb Technol.

[B41] Neumann G, Kabelitz N, Zehnsdorf A, Miltner A, Lippold H, Meyer D, Schmid A, Heipieper HJ (2005). Prediction of the adaptability of *Pseudomonas putida *DOT-T1E to a second phase of a solvent for economically sound two-phase biotransformations. Appl Environ Microbiol.

[B42] Figurski DH, Helinski DR (1979). Replication of an origin-containing derivative of plasmid RK2 dependent on a plasmid function provided in trans. Proc Nat Acad Sci.

